# Positioning with GNSS and 5G: Analysis of Geometric Accuracy in Urban Scenarios

**DOI:** 10.3390/s23042181

**Published:** 2023-02-15

**Authors:** Marianna Alghisi, Ludovico Biagi

**Affiliations:** Dipartimento di Ingegneria Civile Ambientale, Politecnico di Milano, 20133 Milano, Italy

**Keywords:** GNSS and 5G integration, hybrid positioning, precise positioning, urban positioning

## Abstract

GNSS positioning in urban scenarios suffers for the scarce visibility of satellites. Integration with 5G services for positioning could improve this situation. In this paper, the digital surface models (DSMs) relevant to different urban scenarios, namely residential streets and urban canyons, are simulated around one observer in northern Italy (Milano) for one day of the year chosen as an example. The time series of the number of in-view GNSS satellites, their geometry and the derived quality indexes (position dilution of precision (PDOP)) are computed and analyzed. As expected, in urban canyons, a significant number of epochs does not provide four satellites within view, and many more epochs present really mediocre PDOPs. In residential streets, the situation is always quite fair. Different geometric configurations of 5G base stations are simulated around the observer. The availability of 5G times of arrival (ToAs) and their differences (TDoAs) is hypothesized, and the integration of these observations with GNSS pseudoranges is analyzed, again in terms of the PDOPs. In residential streets, 5G availability improves the positioning. In urban canyons, the optimal configuration of 5G base stations (five base stations around the observer) completely solves the positioning problem for all the epochs of the day. Less favorable configurations (four and three base stations) improve epochs with poor PDOPs in a GNSS-only configuration. They allow the positioning of epochs with few satellites but cannot completely replace the GNSS.

## 1. Introduction

Nowadays, positioning and navigation of people and vehicles is a need for society. The computation of precise positions in real time and in single epochs is fundamental for many applications, such as guidance and control, industrial applications, the mass market, public security, assistive technologies and recreational purposes.

In outdoor applications, global navigation satellite systems provide real-time autonomous positioning [1,2]. Standard point positioning in an open field has been considered a globally achieved goal for several years. Processing of multi-frequency pseudoranges can provide accuracies at the meter level. Precise point positioning (PPP) [3] and network geodetic processing of phase observations push accuracy values up to the centimeter or millimeter level [4]. On the contrary, positioning in densely inhabited and built urban environments still remains an open challenge because the scenario affects the accuracy, reliability and even availability of estimates [5]. Buildings, underpasses, moving vehicles and even vegetation block the signals from many satellites, causing poor or even not unsolvable geometries. In addition, reflective surfaces of metal or glass, which are widely present in the urban environment, cause multipathing, which can produce significant errors in low-cost receivers [6]. Note that a reduction in multipathing can be achieved by expensive receivers which do not match the cost requirements of many applications.

Therefore, in an urban scenario, GNSS solutions could be significantly aided by the introduction of additional positioning observations that improve both the geometric configuration and the system redundancy, allowing more reliable and efficient identification and the removal of blunders in observations [7].

The development of hybrid positioning configurations able to integrate a GNSS with other positioning techniques is one of the most popular solutions to overcoming GNSS limitations in harsh environments.

In the past few years, fifth-generation (5G) cellular technology has extended the services and possibilities of use offered by mobile networks in order to meet the needs of an increasingly dynamic and connected society. In particular, 5G, according to the latest release of the Third Partnership Program (3GPP-16), provides highly accurate positioning methods able to compute the precise position of the user with an accuracy at the decimeter level [8,9].

Therefore, 5G positioning methods can be integrated into satellite positioning in a hybrid system in order to improve the quality, availability and reliability of the service.

Among all the possible GNSS processing methods, this paper considers point positioning in a single epoch by undifferenced pseudoranges. In this framework, it describes the advantages that can be obtained through integration with 5G technology. The focus is on the improvement of the geometry and redundancy [10,11].

The accuracy of positioning by time (or distance) observations depends on the geometry of the in-view emitters with respect to the receiver. The aim of this paper is to understand the quality improvements by integrating GNSS pseudoranges with timing observations from a network of 5G base stations. To accomplish this, different urban scenarios and configurations of 5G base stations will be simulated. The relevant values of the positional dilution of precision (PDOP) will be computed and compared. An introduction to GNSS positioning is not needed because many papers and books exist on the topic. On the contrary, an introduction to 5G is proper and is presented in Section 2. The applied working hypotheses and mathematical modeling are discussed in Section 3. The simulated scenarios are described in Section 4, and the results are given in Section 5.

## 2. 5G for Positioning

The diffusion of 5G started in 2019, but the implementation of all its potential is still under study. Referring to the latest official release of the Third Partnership Program (3GPP-16), which formalizes the second phase of 5G, we can say that the advent of 5G officially breaks from previous generations by moving from long-term evolution (LTE) to new radio (NR) technology in order to meet the new communication system requirements dictated by the continuous technological development of new applications.

From a technological point of view, the potential use cases of 5G networks, as described in [12,13], can be summarized in three main usage scenarios:

1. Enhanced mobile broadband (eMBB): This enhanced broadband will supply high speeds for end user data and a high system capacity. This aspect consists of an evolution of the previous LTE technology, improving the connectivity by providing users with enhanced access in densely populated areas both indoors and outdoors. This aspect is not limited to the connection of smartphones; it will improve the connectivity of all devices, promoting the evolution of the Internet of Things (IoT) and the development of smart cities and new services, such as virtual and augmented reality.
2. Massive Machine-Type Communication (mMTC): This is empowered communication between machines over wired or wireless networks with minimal or no human intervention. This scenario is aimed at applications that exchange small volumes of data between a large number of devices, with data typically directed to cloud platforms for subsequent analysis and correlations. The development of this type of communication supports IoT evolution, asset tracking, smart agriculture, smart cities, energy monitoring, smart homes and remote monitoring.
3. Ultra-reliable and low-latency communications (URLLC): This involves applications that have strict reliability and latency requirements that need to be guaranteed. This scenario finds its application in autonomous vehicles, smart grids, remote patient monitoring and telehealth and industrial automation.

The technology of 5G relies on different enabling technologies. Among all the enablers, we have the millimeter wave (mmWave) [14], which consists of an ultra-high-frequency band that ranges from 24.25 GHz to 52.6 GHz (referred to as Frequency 2 (FR2)), in addition to the frequency bands below 6GHz (FR1) which are typical of LTE, according to [15], and are able to solve spectrum scarcity issues in the architecture. Thanks to the bands, it is possible to provide high data rates, an ultra-high capacity and a large bandwidth with low latency. The attenuation of losses is very important because it helps satisfy the demand over urban areas and allows the implementation of new applications. Among all applications, we find the possibility of using mmWave for high-accuracy positioning [16].

According to 3GPP-16, a first change introduced by 5G technology is the reduction of the time base units, which are required to scan the time domain, as shown in Table 1. This has an influence on the achievable improvements in target positioning accuracy.

The technology of 5G provides different positioning observations that can either be uplinked from the user to the base stations or downlinked from the base stations to the user. The different observations can be used in different positioning methods, which are summarized in Table 2 [8,9]. Among all the available techniques, in this paper, we hypothesize working with 5G’s time of arrival (ToA) and time differences of arrival (TDoAs) [17]. In addition, we assume that the 5G base stations are synchronized in time, which is a necessary requirement to fulfill for user positioning. The TDoAs are the differences between two incoming ToAs. To simplify, we can see the ToA as the counterpart of GNSS pseudoranges and TDoAs as single GNSS differences.

Note that from a technical point of view, the 5G positioning architecture provides four different positioning modes, according to the available user equipment and the network organization [9]:

1. User-assisted mode: The user sends his or her observations to a network server that estimates the user positions, and the network provides assistance to the user through location services.
2. User-based mode: With the acquired observations, the user estimates his or her position, and the network provides assistance to the user through location services.
3. Stand-alone mode: The user operates autonomously without any assistance from the network.
4. Network-based mode: The network acquires the observations from the user and estimates his or her position.

However, this point is well beyond the purposes of this paper and and will not be discussed in the following.

In addition, all the above papers mentioned in the references deal with different important aspects of 5G positioning. However, to our knowledge, our paper is the first one in the scientific literature that investigates the geometric improvements obtainable by hybridizing a GNSS and 5G.

## 3. Methods: The Proposed Positioning Model

The proposed analysis relies on the hypothesis that a least squares solution is applied in a single epoch to a set of *m* observations to estimate the position and clock offset of a user. Different configurations of GNSS-alone and GNSS + 5G (ToA and TDoA) will be introduced and discussed.

In a GNSS, the pseudorange observation equation [2] for a receiver *R* and satellite $S_{i}$ expressed in meters is

(1) $$P_{R}^{Si}=\rho_{R}^{Si}+cdt_{R_{GNSS}}+\beta_{R}^{Si}$$

where ρ is the distance between the receiver and the satellite, *c* is the speed of light, $dt_{R_{GNSS}}$ is the clock offset of the receiver with respect to the GNSS reference time and β collects all the other terms that are usually acquired by navigation data or modeled as known (e.g., satellite clock offsets, atmospheric effects and offsets between the different GNSS time scales). The unknowns are the coordinates and the clock offset of the receiver. Actually, a precise approach in GNSS processing should involve a preliminary estimation of the inter-constellation biases in the receiver [18]. However, this is a technical detail outside the scopes of this paper and is not discussed here. In the following, only one, $dt_{R_{GNSS}}$, will be considered an unknown.

In 5G, we can obtain a similar observation equation by multiplying the ToA observation by the speed of light, which is given by

(2) $$ToA_{R}^{i}=\tau_{R}^{BSi}+dt_{R_{5G}}$$

where $\tau_{R}^{BSi}$ is the travel time between the base station and the receiver and $dt_{R_{5G}}$ is the clock offset of the receiver with respect to the 5G network’s time. As in the standard literature, we assumed that the base stations were synchronized within the network and did not present individual clock offsets. Moreover, atmospheric effects were not considered because of the short distance between the receiver and the base stations.

As is typical, the processing of time differences of arrival instead of ToAs was introduced by the 5G scientific and technical literature. The relevant observation equation is

(3) $$TDoA^{i,j}=ToA_{R}^{i}-ToA_{R}^{j}=\tau_{R}^{BSi}-\tau_{R}^{BSj}$$

In the TDoA, the clock offset of the receiver with respect to the 5G network is eliminated because of the single difference.

In order to have a complete analysis of the advantages that could be obtained in terms of positioning quality from the hybridization of 5G and a GNSS, three different configurations were tested:

(1) CG: only GNSS pseudoranges;
(2) CG-TDoA: GNSS pseudoranges + 5G TDoA observations;
(3) CG-ToA: GNSS pseudoranges + 5G ToA observations.

The unknowns to be solved were the following:

(1) CG: the receiver’s coordinates [X,Y,Z] and clock offset with respect to the GNSS time system $dt_{R_{GNSS}}$;
(2) CG-TDoA: the same as for CG.
(3) CG-ToA: aside from the unknowns of CG and CG-TDoA, the receiver’s clock offset with respect to the 5G network $dt_{R_{5G}}$.

As stated before, we tested both the ToA and TDoA in 5G processing, because in the relevant scientific and technical literature, they are equally popular. On the contrary, only undifferenced GNSS pseudoranges are usually processed, and they will be discussed in this paper. Finally, in the following simulations, to harmonize the dimensions of the GNSS and 5G observations, we hypothesized the use of “pseudo” ToAs or TDoAs, which are defined as follows:

(4) $$P_{ToA}=c\times ToA$$

(5) $$P_{TDoA}=c\times TDoA$$

In general, the least squares method requires the linearization of the observations with respect to the unknowns and building of the linear system [19] that relates the observations and unknowns:

(6) $$y_{0}=Ax+\nu$$

(7) E{ν}=0

(8) $$C_{\nu \nu }=\sigma^{2}Q_{yy}$$

The final solution is given by

(9) $$x=N^{-1}A^{T}Q_{yy}^{-1}y_{0}$$

(10) $$N=A^{T}Q_{yy}^{-1}A$$

where *x* is the *n* dimensional unknown vector, $y_{0}$ contains the *m* observations minus the known terms, *A* is the [m×m] design matrix and $Q_{yy}$ is the cofactor matrix of the observations. See the following for our specific case.

The final covariance matrix of the estimates is given by

(11) $$C_{\hat{x}\hat{x}}={\hat{\sigma }}^{2}N_{\hat{x}\hat{x}}^{-1}$$

where ${\hat{\sigma }}^{2}$ is the a posterior variance of the observations.

In our case, the design matrices relevant to the cases of CG, CG-TDoA or CG-ToA are given in Equations (12) and (13):

(12) $$A=\left[\begin{array}{cccc} e_{X}^{S^{1}} & e_{Y}^{S^{1}} & e_{Z}^{S^{1}} & 1\\ e_{X}^{S^{2}} & e_{Y}^{S^{2}} & e_{Z}^{S^{2}} & 1\\ e_{X}^{5G^{1,2}} & e_{Y}^{5G^{1,2}} & e_{Z}^{5G^{1,2}} & 0\\ e_{X}^{5G^{1,3}} & e_{Y}^{5G^{1,3}} & e_{Z}^{5G^{1,3}} & 0\\ \cdots & \cdots & \cdots & 0\\ e_{X}^{5G^{1,k}} & e_{Y}^{5G^{1,k}} & e_{Z}^{5G^{1,k}} & 0 \end{array}\right]$$

(13) $$A=\left[\begin{array}{ccccc} e_{X}^{S^{1}} & e_{Y}^{S^{1}} & e_{Z}^{S^{1}} & 1 & 0\\ e_{X}^{S^{2}} & e_{Y}^{S^{2}} & e_{Z}^{S^{2}} & 1 & 0\\ \cdots & \cdots & \cdots & 1 & 0\\ \cdots & \cdots & \cdots & 1 & 0\\ e_{X}^{S^{n}} & e_{Y}^{S^{n}} & e_{Z}^{S^{n}} & 1 & 0\\ e_{X}^{5G^{1}} & e_{Y}^{5G^{1}} & e_{Z}^{5G^{1}} & 0 & 1\\ e_{X}^{5G^{2}} & e_{Y}^{5G^{2}} & e_{Z}^{5G^{2}} & 0 & 1\\ \cdots & \cdots & \cdots & 0 & 1\\ e_{X}^{5G^{k}} & e_{Y}^{5G^{k}} & e_{Z}^{5G^{k}} & 0 & 1 \end{array}\right]$$

For the GNSS, the first three columns of the design matrix contain the components of the unitary vectors from the satellites to the receiver. For 5G, in the case of $P_{ToA}$, they are the components of the unitary vectors from the base stations to the receiver. For $P_{TDoA}$, they are the differences between the above unitary vectors.

The fourth column of the design matrix contains the coefficient that multiplies the GNSS clock offset of the receiver. The fifth column, present only in CG-ToA, contains the 5G clock offset coefficients.

$Q_{yy}$ is the cofactor matrix of the observations and has dimensions of [m×m]. In our simulation, we assumed that the GNSS pseudoranges and 5G ToA were uncorrelated and had the same accuracy. The cofactor matrix of the GNSS pseudoranges and 5G $P_{ToA}$ was the [m×m] identity.

Clearly, the introduced assumption of equal accuracy for 5G and GNSS observations may be considered simplistic. This choice was made according to the standards provided by the 5G technical literature, which aim to reach the decimeter level in terms of positioning accuracy. At present, the first 5G positioning models based on simulated [20] and synthesized 5G observations [21] meet the standards advanced by the literature, although solutions based on experimental data obtained from campaigns or field measurements are not available yet.

Consequently, the block of the covariance matrix of the TDoAs can be suitably computed using the following propagation law:

(14) $$Q_{TDoA}=T\times I\times T^{T}$$

where *I* is the identity cofactor matrix of ToA observations and *T* is the transformation matrix to obtain the TDoAs from the ToA observations:

(15) $$T=\left[\begin{array}{ccccc} 1 & -1 & 0 & \cdots & 0\\ 1 & 0 & -1 & \cdots & 0\\ \cdots & \cdots & \cdots & \cdots & \cdots \\ 1 & \cdots & \cdots & \cdots & -1 \end{array}\right]$$

The positions of the satellites can be computed using the ephemerides. The positions of the 5G base stations and the receiver are given by the simulation choices. Therefore, the design matrix can be computed, and consequently, the PDOP can be derived:

(16) $$PDOP=q_{1,1}+q_{2,2}+q_{3,3}$$

where $q_{i,i}$ represents the diagonal elements on $N^{-1}$. The adopted classification values are shown in Table 3.

Clearly, if the coordinates are expressed in a local Cartesian east, north, up system, then $q_{1,1}$, $q_{2,2}$ and $q_{3,3}$ are the accuracy indexes for east, north and up, respectively. Such a separation allows individually analyzing the horizontal and vertical accuracies. This is useful because horizontal navigation is often a strict requirement in urban scenarios.

## 4. Simulation

The urban simulation was carried out through four different scenarios that could represent the setting of a plain city such as Milan well. Each scenario represented an urban street with a width of 9 m, comprising a dual carriageway and sidewalks on both sides of the street. The heights of the buildings facing the street were constant. In the implementation, two possible building heights were adopted: 24 m to simulate urban canyons and 9 m to simulate residential streets.

The developed scenarios were the following:

1. EW-24: street from east to west with buildings 24 m high;
2. EW-9: street from east to west with buildings 9 m high;
3. NS-24: street from north to south with buildings 24 m high;
4. NS-9: street from north to south with buildings 9 m high.

Each street had a total extension of 20 km in order to numerically represent an infinitely extended road from −10 up to +10 km with respect to the receiver that was placed in a conventional position within Politecnico di Milano on the GRS80 ellipsoid surface (latitude $45^{\circ }28^{\prime }42^{\prime \prime }$ N, longitude $9^{\circ }13^{\prime }45^{\prime \prime }$ E, hellipsoidal height = 160 m). The buildings were implemented with a grid of points around the observer.

### 4.1. GNSS Satellite Visibility

**Figure 1:**
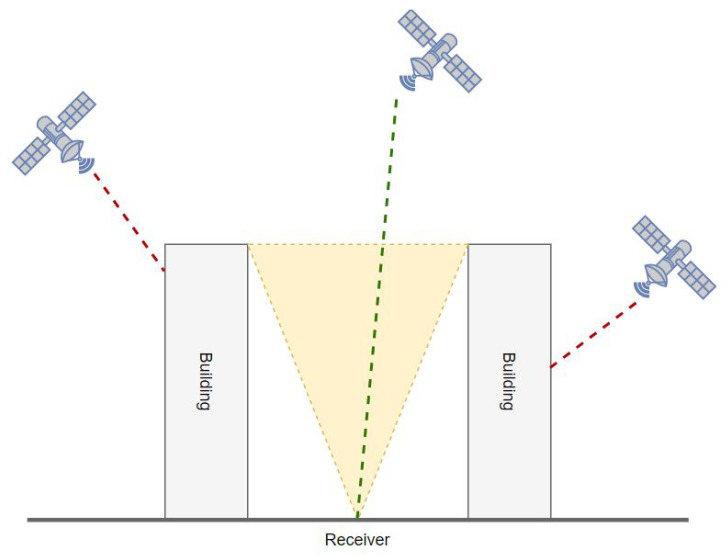
Satellites in view and not in view in urban environment.

We developed an algorithm that identifies the in-view GNSS satellites for each simulated scenario. We assumed that the receiver was able to obtain signals from four different constellations: GPS, Galileo, Glonass and BeiDou. The satellite positions were retrieved from the ESA’s precise ephemerides for one example day, namely 6 May 2022. At each epoch of the day (one epoch every 5 min), the algorithm computed the azimuth and elevation of each satellite with respect to the observer. The elevations and azimuths were used to select satellites that were actually in view (i.e., above the grid of simulated buildings) (see Figure 1).

### 4.2. 5G Network Simulation

**Figure 2:**
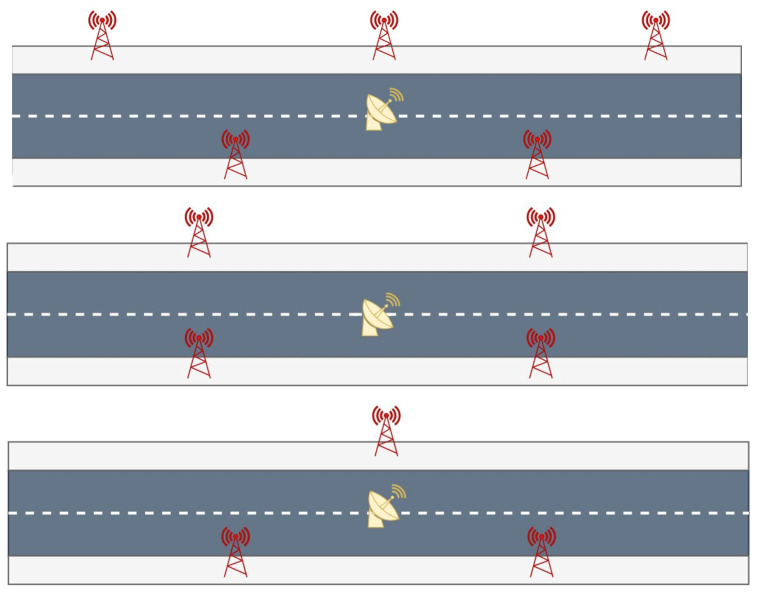
Possible network configurations. In order from the top: N5 (five staggered base stations), N4 (four paired base stations) and N3 (three staggered base stations).

According to the literature, 5G base stations were introduced in each scenario at roadside on both sides of the street at an equal spacing of 200 m. Their heights were set to 20 m. The simulation was carried out by testing three possible 5G network configurations differentiated by the number and geometries of the base stations around the observer. The networks are represented in Figure 2:

(1) Network N5: five staggered base stations, with one every 100 m on alternate sides of the street;
(2) Network N4: four mirrored base stations, with the receiver exactly in the center;
(3) Network N3: three staggered base stations.

## 5. Results

**Figure 3:**
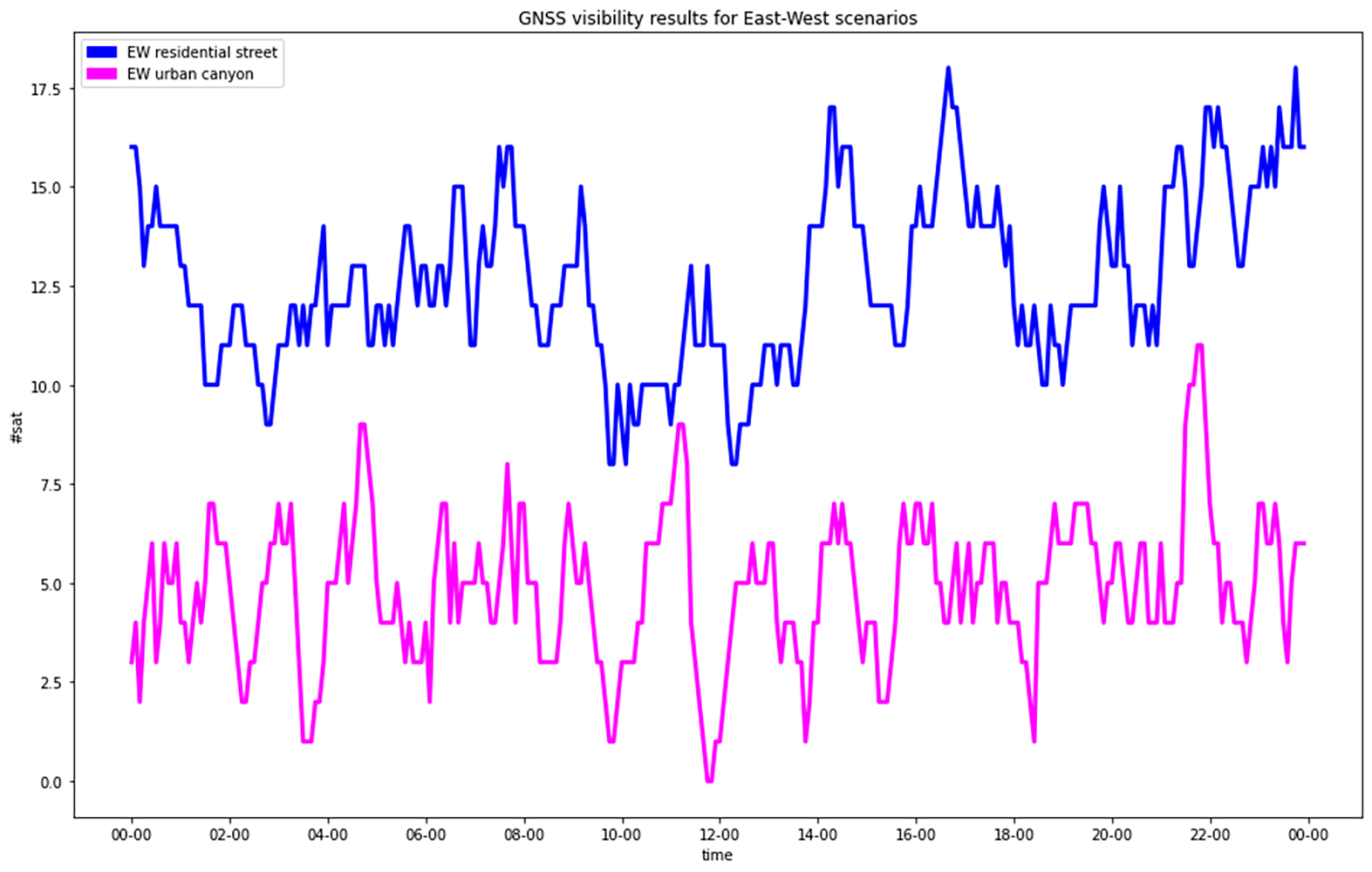
GNSS visibility for east-west streets (blue = RA; pink = UC).

**Figure 4:**
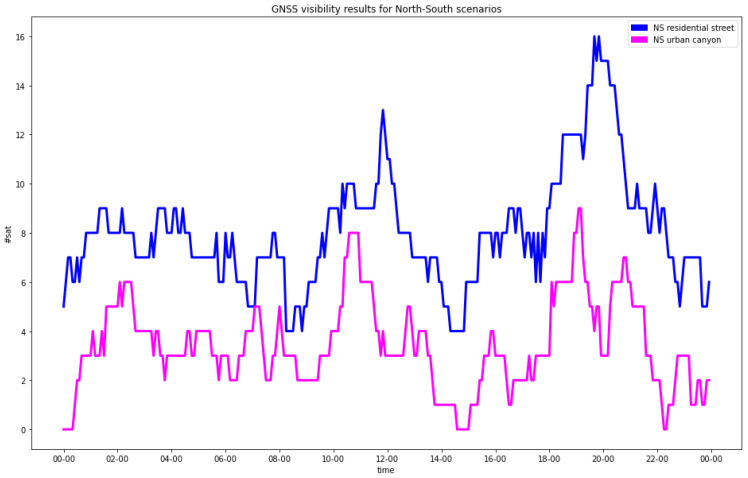
GNSS visibility for north-south streets (blue = RA; pink = UC).

The analysis of the satellites’ visibility confirmed what was expected: the presence of buildings caused a clear degradation of the satellites’ configuration (Table 4 and Figure 3 and Figure 4). In the residential streets, a general decrease in the number of visible satellites was observed, and the NS street provided worse statistics than the EW one. In any case, all the epochs provided at least four visible satellites, which was the minimal condition to estimate the positions.

In the urban canyons, for a significant number of epochs (61 for the EW street and 172 for the NS street out of 288), less than 4 satellites were in view.

Once the visibility of the satellites was obtained, the PDOP indexes were computed for each scenario and for all the possible configurations: GNSS-alone (CG), GNSS + TDoA. (CG-TDoA) and GNSS + ToA (CG-ToA). In CG, the PDOP could be computed only by the GNSS and therefore only for epochs with at least four in-view satellites. In CG-TDoA and CG-ToA, the PDOP was computed by also considering the 5G base stations.

First, let us analyze the pure 5G configuration, which is relevant to epochs with no visible GNSS satellites. Theoretically, both the N5 and N4 networks guaranteed enough observations for positioning (five and four ToAs or four and three TDoAs, respectively). However, as we will discuss in Section 5.2 the specific geometry simulated for N4 generated an ill-conditioned system that could not be inverted and solved. Therefore, the specific geometry of N4 is not suitable for positioning purposes. Three 5G base stations ( network N3) provided fewer observations than the unknowns and could not be used without a GNSS to estimate the positions.

### 5.1. N5: Hybrid GNSS+5G with Five Base Stations

In the following section, the results related to the N5 5G network are presented and compared.

**Figure 5:**
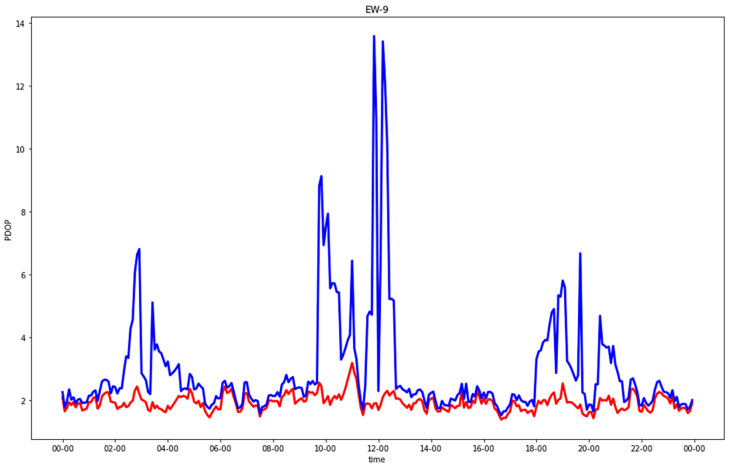
PDOP in east-west residential street for GNSS-alone (blue) compared with GNSS + five 5G stations (red).

**Figure 6:**
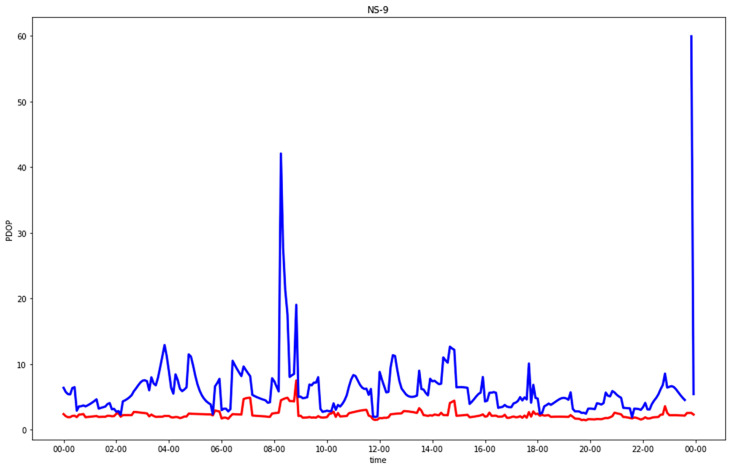
PDOP in north-south residential street for GNSS-alone (blue) compared with GNSS + five 5G stations (red), plotting only results with PDOP < 100.

**Figure 7:**
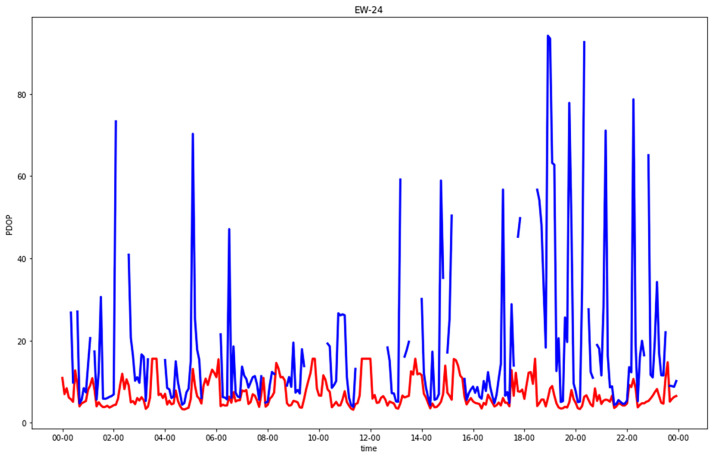
PDOP in east-west urban canyon for GNSS-alone (blue) compared with GNSS + five 5G stations (red), plotting only results with PDOP < 100.

**Figure 8:**
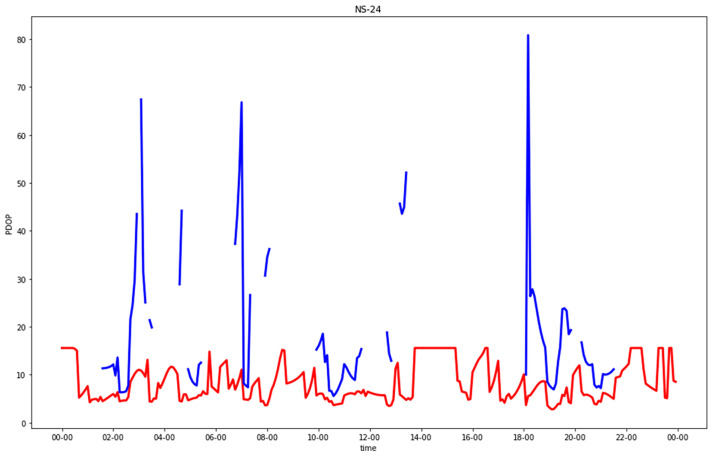
PDOP in north-south urban canyon for GNSS-alone compared with GNSS + five 5G stations, plotting only results with PDOP < 100.

Figure 5, Figure 6, Figure 7 and Figure 8 show comparisons of the PDOP values for the GNSS-only and hybrid configurations in the four urban scenarios. Note that in the plots, to improve the graphical rendering, PDOPs greater than 100 were not plotted. Table 5 and Table 6 show the statistics.

Focusing on urban canyons, in GNSS-alone (CG), two kinds of “bad” epochs existed: either less than four satellites in view or at least four satellites but with a bad geometry with respect to the observer. In the former case, the PDOP was infinite, and in the latter case, a solution could be numerically computed, but its accuracy was not acceptable. The EW streets provided better solutions than the NS ones. The separate analysis of horizontal and vertical DOPs, which was not graphically reported, highlighted something that could be expected: the degrading in many epochs of the PDOP due to the horizonal cooordinate orthogonal to the street direction (i.e., north in EW streets or east in NS streets), which was in the absolute worst estimable, even with respect to the up coordinate.

The introduction of 5G significantly improved all the solutions, leading to a maximum PDOP equal to 9.4, which corresponded to epochs without (or with just one) visible satellites. Such a value in the standard scale (Table 3) can be defined as “moderate”. The results were quite excellent for both the NS and EW streets.

In the residential streets, GNSS-alone already provided fair results for the EW street and those which were a little worse for the NS street. Even in this case, the hybrid solution improved the worst epochs significantly.

**Figure 9:**
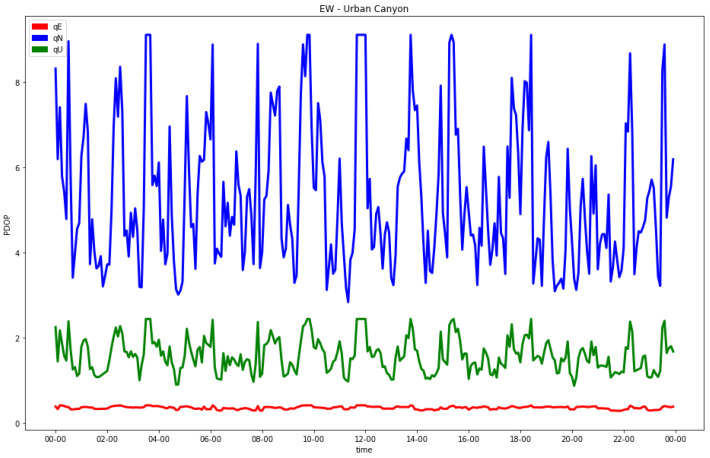
Diagonal coefficients in N (blue), E (red) and U (green) coordinates of final co-factor matrix in EW street (C5N5-CG-TDoA) for urban canyon.

**Figure 10:**
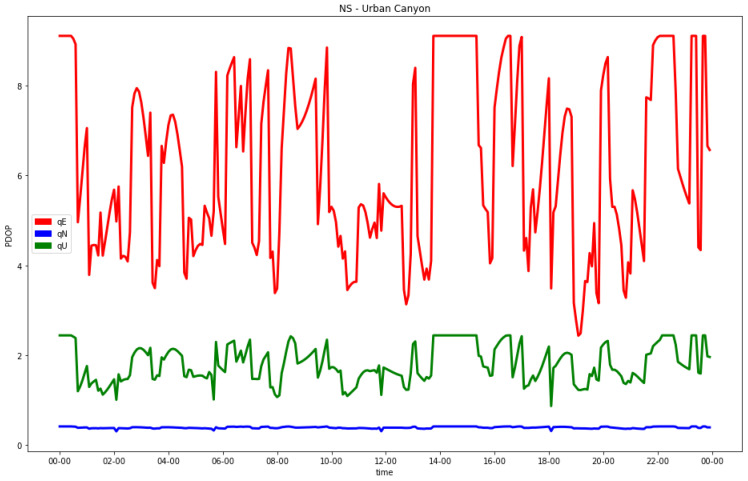
Diagonal coefficients in N (blue), E (red) and U (green) coordinates of final co-factor matrix in NS street (C5N5-CG-TDoA) for urban canyon.

In addition, for the hydrid GNSS and 5G configuration, the individual analysis of the indexes in the local east, north and up coordinates were performed. In this case, the worst estimable coordinate was also the horizontal one orthogonal to the direction of the street. As an example, the results of the EW and NS urban canyons with the C5N5-CG-TDoA signal configuration are shown in Figure 9 and Figure 10, respectively.

A further consideration was made regarding the horizontal DOP (HDOP) and vertical DOP (VDOP) indices. As expected, the values obtained for the HDOPs in the C5N5-CG configuration did not differ significantly from the respective PDOPs. Indeed, as previously stated, the index was mainly driven by the horizontal value orthogonal to the direction of the road.

The statistics of the results provided by ToA processing (CG-ToA) are shown in Table 7 and can be compared with those previously discussed for the TDoA. The quality of the CG-ToA results was comparable to the CG-TDoA results but slightly worse in both the urban canyons and residential streets. The difference was not significant, but it was not expected.

### 5.2. N4: Hybrid GNSS and 5G with Four Base Stations

First of all, we decided to verify if the configuration with staggered base stations was the optimal one, comparing it with a configuration of four mirrored base stations (N4) as shown in Figure 2. In this case, the PDOPs were also computed for each epoch and for both the TDoA and ToA.

As previously stated, four base stations provided the needed number of observations to solve the system even in the case where no GNSS was available. On the contrary, in such epochs (Table 8), positioning was impossible. This was caused by the poor conditioning of the specific geometry of N4. In particular, the height represented an inestimable coordinate.

Different positions of the user with respect to the four base stations were investigated. Even when moving the receiver away from the center of the 5G network, the solution system remained poorly conditioned, with PDOP values that exceed tens of thousands.

The poor conditioning remained with one or two in-view satellites as well. The PDOP diverged, reaching maximum values to the order of magnitude of $10^{12}$. For epochs with three or more satellites, as expected, the introduction of 5G base stations improved the results.

To verify that the poor conditioning was caused by the geometry of the base stations, we repeated the analysis with a network of four staggered base stations. The results obtained were similar to those of N5, with the PDOP values being just slightly greater.

The same results are reported in Table 9 for the residential scenarios. The results slightly improved with respect to the GNSS-alone solutions.

### 5.3. N3: Hybrid GNSS+5G and Three 5G Base Stations

The last tested network contained three staggered base stations around the observer. This solution can represent the typical low-coverage urban environment network well. In epochs with no or one in-view satellite, the PDOP could not be computed, and the positions could not be estimated. Table 10 and Table 11 report the obtained results for urban canyons and residential streets, respectively. Despite the missing epochs with less than two satellites, the results were fair. In any case, some epochs with very poor GNSS geometries still presented high PDOP values. In addition, the results became worse for the north-south street direction, as previously noticed.

### 5.4. Final Considerations for Positioning Quality

**Figure 11:**
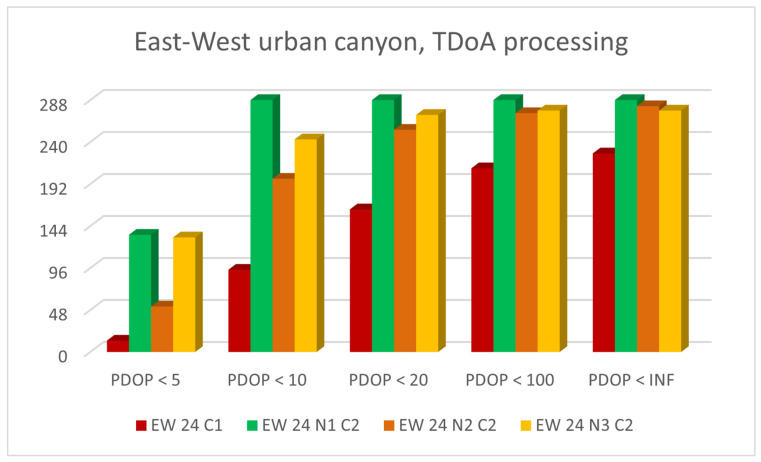
Number of epochs with PDOP values smaller than 5, 10, 20, 100 or *∞* for east-west urban canyon with CG, N5-CG-TDoA, N4-CG-TDoA and N3-CG-ToA signal configurations.

**Figure 12:**
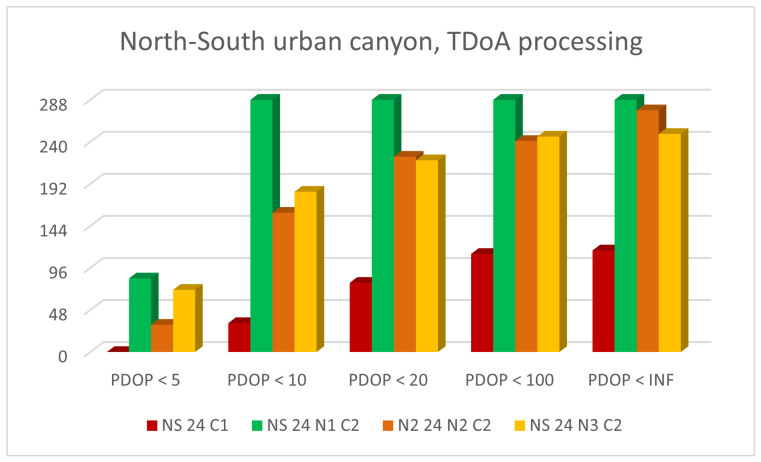
Number of epochs whit PDOP values smaller than 5, 10, 20, 100 or *∞* for north-south urban canyon with CG, N5-CG-TDoA, N4-CG-TDoA and N3-CG-ToA signal configurations.

Figure 11 and Figure 12 show histogram charts of the number of epochs classified by the criteria presented in Table 3: a PDOP value smaller than 5, 10, 20, 100 or *∞*.

The figures show only the results obtained for urban canyons, since these were the most interesting and critical subject. The GNSS was only compared with the results obtained when integrating TDoA for the N5, N4 and N3 networks. The results obtained using the ToA were similar.

N5 was optimal, since all the epochs presented a fair PDOP. N4 was the worst network due to the poor geometry of the four base stations.

This section may be divided by subheading. This should provide a concise and precise description of the experimental results, their interpretation as well as the experimental conclusions that can be drawn.

## 6. Conclusions

The DSMs relevant to different urban scenarios, namely residential streets and urban canyons, were simulated around an observer in northern Italy for a day of the year. The time series of the number of in-view GNSS satellites and the derived PDOPs were computed and analyzed. Then, the integration of GNSS pseudoranges by 5G ToA and TDoA was investigated for different geometric configurations of 5G base stations. As expected, the quality of the GNSS-alone positioning was quite fair in residential streets, while the results in urban canyons suffered from obstructions. For the east-west and north-south streets, 20% and 60% of the epochs simply did not provide enough observations, respectively, while many others presented quite mediocre PDOPs. In urban canyons, an optimal configuration of 5G base stations (five around the observer) solved all the problems. Note that in such a configuration, 5G by itself would guarantee estimating the positions. In any case, the integration of a GNSS and 5G increased both the quality and the redundancy of the solutions. Less ideal configurations of 5G base stations are not self sufficient and cannot solve the worst epochs. In any case, they can improve the quality of mediocre GNSS epochs and again increase the redundancy of the system, which in principle could help in outlier rejection. For the residential streets, integration of a GNSS and 5G always improved the solutions. Moreover, the number of 5G base stations was not the only parameter to influence the quality of the final results; their geometry was also a fundamental factor that affected the final results. Lastly, according to the results, ToA and TDoA processing provided almost the same quality, but in any case, the TDoA seemed to be slightly better than the ToA.

The next step of our work will focus on evaluation of the actual accuracy of the available 5G observations and analysis of the actual synchronization condition of the networks through the acquisition and processing of real 5G data which are not synthesized or simulated. These procedures will take place in the framework of a Hybrid Positioning Engine Running on 5G and GNSS (HYper 5G), an ESA NAVISP program project which aims at studying, designing and developing the algorithms and software needed to implement a precise positioning engine that jointly uses multi-constellation GNSS and 5G observations.

## Figures and Tables

**Table 1:** Comparison between the time base units (required to represent the time domain) of LTE and new radio (5G) technologies. We refer to the LTE time base unit as $T_{s}$ and to 5G’s as $T_{c}$. The second column reports Ts and Tc in seconds, and in the third, they are converted into meters. $\Delta f_{max}$ is the subcarrier spacing (SCS), and $N_{f}$ is the number of Fourier points.

	Time Base Unit $T=\frac{1}{\Delta f_{max}\cdot N_{f}}$	Distance d=cT
LTE	$T_{s}=\frac{1}{15000\cdot 2048}≅32.55\;\mathrm{ns}$	$d_{s}=10\;m$
5G	$T_{c}=\frac{1}{48000\cdot 4096}≅0.51\;\mathrm{ns}$	$d_{c}=15\;\mathrm{cm}$

**Table 2:** Available 5G positioning methods [8,9].

Method	Description
DL-TDOA: Downlink Time Difference of Arrival	Based on Time of Arrival (TOA) measurements of DL signals received from multiple base stations (BSs) to user equipment (User). Computed quantities: - OTDOA: Observed TDOA - RTD: Real time difference - GTD: Geometric time difference
DL-AOD: Downlink Angle of Departure	Based on reference signal received power (RSRP) measurements performed by user. User requires assistance data from the network: a list of candidate BSs, BSs’ geographical locations and beam information.
UL-TDOA: Uplink Time Difference of Arrival	User’s signal is received by multiple BSs, which compute the TOA. Measurements have a common time scale and are called UL-Relative TOA (UL-RTOA). Then, they are sent to the location management function (LMF), which computes the TDOA.
UL-AOA: Uplink Angle of Arrival	The received signal from the user is transformed by gNodeB (gNB) in azimuth and elevation, and directional antennas are required, implying the network-based mode.
RTT: Round Trip Time	Uses two-way TOA measurements and requires no BS synchronization.
MC RTT: Multi-Cell Round Trip Time	Estimate RTT between multiple gNBs, requires both UL and DL. No synchronization errors.

**Table 3:** Reference values for DOP indexes.

Value	Class
1–5	Good
5–10	Moderate
10–20	Fair
>20	Poor

**Table 4:** Statistics of visible GNSS satellites in urban scenarios compared with open sky conditions. EW = east-west streets; NS = north-south streets; UC = urban canyon (24-m-high buildings); RS = residential street (9-m-high buildings); N4 = number of epochs with nsat<4 out of 288 epochs.

	Open Sky	EW-UC	NS-UC	EW-RS	NS-RS
Mean	40	5	3	13	8
Min	30	0	0	8	4
Max	47	11	9	18	9
StDev	3.5	1.8	1.8	2.1	2.3
N4	/	61	172	0	0

**Table 5:** Urban canyon (24-m-high buildings) scenarios: statistical analysis of the PDOP for GNSS-alone (CG) and GNSS + TDoA from five 5G base stations (CG-TDoA/N5). EW = east-west street; NS = north-south street.

	EW CG	EW CG-TDoA/N5	NS CG	NS CG-TDoA/N5
Count	227/288	288	116/288	288
Mean	57	5.5	27	6.5
Min	3.9	3.0	5.6	2.8
Max	3189	9.4	681	9.4
StdDev	235	1.7	65	1.9

**Table 6:** Residential street (9-m-high buildings) scenarios: statistical analysis of the PDOP for GNSS-alone (CG) and GNSS + TDoA from five 5G base stations (CG-TDoA/N5). EW = east-west street; NS = north-south street.

	EW CG	EW CG-TDoA/N5	NS CG	NS CG-TDoA/N5
Count	288	288	288	288
Mean	2.9	1.8	9.0	2.2
Min	1.5	1.3	1.9	1.3
Max	13.6	3.1	752	5.6
StdDev	1.7	0.3	44.7	0.6

**Table 7:** PDOP statistical analysis for GNSS + five 5G stations in case of ToA processing (EW = east-west; NS = north-south; UC = urban canyon; RS = residential street.

	EW-UC	NS-UC	EW-RS	NS-RS
Count	288	288	288	288
Mean	6.1	7.1	1.9	2.2
Min	3.1	2.8	1.4	1.4
Max	11	11	3.1	6.5
Std	2.2	2.5	0.25	0.65

**Table 8:** Urban canyon scenarios: statistical analysis of PDOP for GNSS and four mirrored 5G stations (N4) in TDoA (CG-TDoA) and ToA (CG-ToA) processing.

East-West Urban Canyon		
	N4/CG-TDoA	N4/CG-ToA
Count	281/288	278/288
Mean	$1.2\cdot 10^{10}$	$1.4\cdot 10^{6}$
Min	3.4	3.4
Max	$1.6\cdot 10^{12}$	$2.2\cdot 10^{8}$
StdDev	$1.4\cdot 10^{11}$	$1.6\cdot 10^{7}$
**North-South Urban Canyon**		
	N4/CG-TDoA	N4/CG-ToA
Count	276/288	265/288
Mean	$1.9\cdot 10^{11}$	$6.0\cdot 10^{6}$
Min	3.4	3.4
Max	$4.0\cdot 10^{12}$	$2.4\cdot 10^{8}$
StdDev	$8.8\cdot 10^{11}$	$2.6\cdot 10^{7}$

**Table 9:** Residential streets: statistical analysis of PDOP for GNSS and four mirrored 5G stations (N4) in TDoA (CG-TDoA) and ToA (CG-ToA) processing.

East-West Residential Streets		
	N4/CG-TDoA	N4/CG-ToA
Count	288	288
Mean	2.5	2.6
Min	1.5	1.5
Max	12	12
StdDev	1.2	1.3
**North-South Residential Streets**		
	N4-CG-TDoA	N4-CG-ToA
Count	288	288
Mean	3.4	3.5
Min	1.7	1.8
Max	11	12
StdDev	1.6	1.6

**Table 10:** Urban canyons: statistical analysis of PDOP for GNSS and three staggered 5G stations (N3) in TDoA (CG-TDoA) abd ToA (CG-ToA) processing.

East-West Urban Canyon		
	N3-CG-TDoA	N3-CG-ToA
Count	276/288	276/288
Men	6.6	6.9
Min	3.0	3.0
Max	73	73
Std	6.3	6.4
**North-South Urban Canyon**		
	N3-CG-TDoA	N3-CG-ToA
Count	249/288	249/288
Mean	14.4	14.6
Min	2.7	2.8
Max	500	500
StdDev	41	41

**Table 11:** Residential streets: statistical analysis of PDOP for GNSS and three staggered 5G stations (N3) in TDoA (CG-TDoA) and ToA (CG-ToA) processing.

East-West Residential Streets		
	N3-CG-TDoA	N3-CG-ToA
Count	288	288
Mean	1.9	1.9
Min	1.3	1.4
Max	3.7	3.7
StdDev	0.3	0.3
**North-South Residential Streets**		
	N3-CG-TDoA	N3-CG-ToA
Count	288	288
Mean	2.3	2.4
Min	1.4	1.5
Max	7.9	7.9
Std	0.8	0.8

## Abbreviations

GNSS	Global navigation satellite system
5G	Fifth generation of mobile communication technology
DSM	Digital surface model
PDOP	Positional dilution of precision
HDOP	Horizontal dilution of precision
VDOP	Vertical dilution of precision
ToA	Time of arrival
TDoA	Time difference of arrival
3GPP-16	Sixteenth release of the Third Partnership Program
LTE	Long-term evolution
NR	New radio
eMBB	Enhanced mobile broadband
IoT	Internet of Things
mMTC	Massive Machine-Type Communication
URLLC	Ultra-reliable and low-latency communications
CG	Configuration GNSS pseudoranges only
CG-TDoA	Configuration GNSS pseudoranges + 5G TDoA
CG-ToA	Configuration GNSS pseudoranges + 5G ToA
EW-UC	Street from east to west with buildings 24 m high
EW-RA	Street from east to west with buildings 9 m high
NS-UC	Street from north to south with buildings 24 m high
NS-RA	Street from north to south with buildings 9 m high
N5	5G network of five staggered base stations
N4	5G network of four mirrored base stations
N3	Network with three staggered base stations
